# Effect of Soybean Meal Substitution by Raw Chickpea Seeds on Thermal Properties and Fatty Acid Composition of Subcutaneous Fat Tissue of Broiler Chickens

**DOI:** 10.3390/ani10030533

**Published:** 2020-03-22

**Authors:** Waldemar Paszkiewicz, Siemowit Muszyński, Małgorzata Kwiecień, Mykola Zhyla, Sylwester Świątkiewicz, Anna Arczewska-Włosek, Ewa Tomaszewska

**Affiliations:** 1 Department of Food Hygiene of Animal Origin, Faculty of Veterinary Medicine, University of Life Sciences in Lublin, Akademicka St. 12, 20-950 Lublin, Poland; waldemar.paszkiewicz@up.lublin.pl; 2 Department of Biophysics, Faculty of Environmental Biology, University of Life Sciences in Lublin, Akademicka St. 13, 20-950 Lublin, Poland; 3 Institute of Animal Nutrition and Bromatology, Faculty of Animal Sciences and Bioeconomy, University of Life Sciences in Lublin, Akademicka St. 13, 20-950 Lublin, Poland; malgorzata.kwiecien@up.lublin.pl; 4 Laboratory of Clinical Biological Research, State Scientific Research Control Institute of Veterinary Medicinal Products and Feed Additives, Donetska St. 11, 79000 Lviv, Ukraine; zhyla-m@ukr.net; 5 Department of Animal Nutrition and Feed Science, National Research Institute of Animal Production, Krakowska St. 1, 32-083 Balice, Poland; s.swiatkiewicz@izoo.krakow.pl (S.Ś.); anna.arczewska@izoo.krakow.pl (A.A.-W.); 6 Department of Animal Physiology, Faculty of Veterinary Medicine, University of Life Sciences in Lublin, Akademicka St. 12, 20-950 Lublin, Poland; ewaRST@interia.pl

**Keywords:** chickpea, broiler chickens, subcutaneous fat tissue, thermal analysis

## Abstract

**Simple Summary:**

A soybean meal, commonly used as a primary source of protein in animal diets, is often obtained from processing genetically modified soybean varieties. The tendency to reduce the use of feeds containing transgenic constituents increases the need for alternative sources of dietary protein. In this study, we fed broiler chickens diets containing either soybean meal or raw chickpea seeds. We examined the effect of such a substitution on broilers’ subcutaneous fat tissue, which has numerous important physiological functions in chickens.

**Abstract:**

In this study, the effect of soybean meal substitution by raw chickpea seeds on the thermal properties and fatty acid profile of subcutaneous fat tissue of broiler chickens was examined. The experiment, performed on Ross 308 chickens, lasted for 42 days. Tight subcutaneous fat tissue was analyzed using differential scanning calorimetry (DSC) measurements while the fatty acid composition of subcutaneous adipose tissue was determined chromatographically. There was no effect of soybean meal substitution on fat crystallization temperature or crystallization enthalpy. However, the total calorimetric enthalpy of the melting of low-melting monounsaturated and saturated triacylglycerols differed between groups. Fatty acid proportions in the subcutaneous fat tissue of broiler chickens were also altered. Among others, chickpea seed inclusion decreased the content of main saturated acid (palmitic acid) and increased the content of main monounsaturated (oleic) and tri-unsaturated (linolenic) acids. The results show that the soybean meal substitution by raw chickpea seeds in the feed can affect the structural properties of adipose tissue in broiler chickens, including the thermal transformation of unsaturated fatty acids. Due to the numerous physiological functions of subcutaneous fat tissue, understanding these mechanisms can promote the use of alternative protein both in poultry and human nutrition.

## 1. Introduction

Adipose tissue, although undesirable from the point of view of meat processing and consumer acceptance, has important physiological functions in animal organisms. In the carcasses of chicken broilers, subcutaneous fat accounts for 11–18% of total body weight [1,2,3] and almost one third of subcutaneous fat is located in the pelvic limbs [4]. Subcutaneous fat tissue in the pelvic limbs provides thermal insulation of the external envelope of the body, serves as a source of energy for muscles, and has immunomodulatory and metabolic roles [5]. Next to its genetic background, nutrition is the most important factor influencing the composition and quality characteristics of fat tissue in broilers [6]. The amount and quality of fat and protein in the feed play an important part, not only in the regulation of the overall lipid metabolism in the bird’s body, but also in the characteristics of subcutaneous fat [1,2,3]. A soybean meal (SBM), commonly used as a primary source of protein in animal diets, is often obtained from processing genetically modified soybean varieties. The tendency to reduce the use of feed containing GMOs (genetically modified organisms), and the growing customer preferences for animal products obtained from animals not given such feeds, increase the need to look for alternative sources of dietary protein. Previous studies indicate that alternatives to SBM can be faba bean [7,8], lupines [9,10,11], chickpeas [12,13], and, as it was found more recently, nonplant protein sources such as insect meal [14,15,16]. As compared to soybean, chickpea seeds (CPS) are characterized by a lower content of chymotrypsin and trypsin inhibitors, while the nutritional value of chickpea protein is comparable to that of soybean [17]. However, antinutritive factors like protease inhibitors, phytates, and nonstarch polysaccharides present in unprocessed raw seeds have adverse effects on nutrient digestibility and can lead to a reduction of absorption of nutrients [17]. Compared to chickpea seeds, soybean meal contains also more fiber which improves the retention of soluble ash and increases the production of hydrochloric acid, improving the solubility of mineral salts.

Differential scanning calorimetry (DSC) is a thermal analysis technique which allows the detection of conformational changes associated with phase transitions of proteins and lipids forming the structure of the examined tissues. It is a fast and environmentally friendly technique, as it does not require the use of chemical reagents. In medical sciences, there are reports showing that DSC analyses can complement histological or biochemical analyses, both in experimental works [18,19] and, potentially, in diagnostics where it has been acknowledged as a novel tool for diagnosing and monitoring several diseases [20,21,22,23,24]. In recent years, DSC is also becoming a more widely used method for analyzing animal tissues and food of animal origin [25,26,27]. DSC analysis can be used for determining the crystallization temperature of processed fat, and the melting point of fractions of triacylglycerols (TAGs), which have the greatest impact on the thermal parameters of adipose tissue [5,28,29,30].

In our previous studies, we have examined the effects of CPS as a substitute of SBM in broiler diets on selected properties of broiler muscles [31], bones [13], tendons [12], and skin tissue [32]. The aim of these research investigations was to increase our understanding about how broiler chickens respond to a dietary inclusion of raw, unprocessed CPS.

As subcutaneous fat tissue has important physiological functions, the aim of the present study was to determine, by the means of DSC analysis, the thermal properties of subcutaneous fat tissue of broilers fed raw CPS as a substitution of SBM. Both diets were only adjusted to be isoprotein, isonitrogenous and isoenergetic, without additional adjustment of other nutrients, including fats. As DSC analysis of thermal properties of broilers tissues is a relatively new concept, the fatty acid (FA) composition of subcutaneous adipose tissue was also determined.

## 2. Materials and Methods

### 2.1. Ethical Approval

All procedures conducted with the chickens had been prior approved by the 2nd Local Ethics Committee for Animal Testing at the University of Life Sciences in Lublin, Poland (33/2015).

### 2.2. Animals and Experimental Design

A total of 160, one-day-old, male, broiler chickens (Ross 308) were used in this experiment. The chicks were randomly allocated into two groups (n = 80 in each), divided into 4 pens (20 birds per pen): the control group (the SBM group), and the experimental group, where soybean meal was replaced by raw chickpea seeds throughout the whole rearing period (the CPS group). The diets were isonitrogenous, isoprotein, and isoenergetic (Table 1). Bird management and care are described in detail in previous works [12,13]. Individual body weight and feed intake (per pen) were monitored. At the 42nd day, 8 birds from each group (2 birds form each replicate pen) were stunned, using the electrical stunning method, and then decapitated. Immediately after slaughter, samples of subcutaneous fat were taken from the same region of the thighs, and stored at 4 ˚C for 24 h.

### 2.3. DSC Measurements

DSC analysis was performed using a Q200 calorimeter (TA Instruments, New Castle, DE, USA) calibrated using the indium standards (melting temperature 156.6 °C, ΔH = 28.45 J/g). Nitrogen (99.999% purity) was the purge gas, and flowed at 50 mL/min. The specimens weighing 8-12 mg were weighted into 40 μL aluminum DSC pans, and hermetically sealed to prevent any loss of moisture during the measurements. An empty, hermetically sealed, aluminium pan was used as reference. The samples were subjected to the following temperature program: a 30 °C isotherm for 5 min, cooled from 30 to −80 °C at a rate of 10 °C/min, −80 °C isotherm for 5 min, heated from −80 to 80 °C at a rate of 10 °C/min. After the analyses, punctured pans were dried for 24 h at 105 °C to determine sample moisture content [33]. The manufacturer’s software (TA Instruments Operating Software) program, integrated with the calorimeter, was used to analyse and plot the thermal data. For each transition peak of crystallization and melting of the lipids, peak temperatures at maximum heat absorption T (°C), and the net enthalpy ΔH (J/g) of thermal transition, were determined. The material collected from each individual was analyzed in duplicate, and the average was calculated. Transition peaks were identified referring to data available in other studies [5,20,34,35].

### 2.4. Determination of Fatty Acid Composition in Subcutaneous Fat

The percentage of fatty acid methyl esters in subcutaneous fat was estimated with gas chromatography on a CP-3800 gas chromatograph (Varian, Palo Alto, CA, USA) after previous extraction of fat with Folch’s method [36]. Gas chromatography was performed using Supelco 37 FAME Mix 47885-U standards (Sigma-Aldrich, St. Louis, MO, USA), with chromatograph operating conditions as follows: the capillary column CP WAX 52CB, DF 0.25 mm x 100 m, flow rate of gas carrier (helium) 1.4 mL/min, column temperature 120 °C gradually increasing by 2 °C/min up to 210 °C, determination time 120 min, feeder temperature 160 °C, detector FID temperature 160 °C, other gases: hydrogen and oxygen. Fatty acids were expressed as a percentage of total fatty acids.

### 2.5. Statistical Analysis

The statistical analyses of the data were performed using Statistica 12 software (TIBCO Software Inc., Palo Alto, CA, USA). The normality of data distribution was tested using the Shapiro–Wilk test. A comparison between normally distributed variables was carried out using the Student’s t test. When the variables were not normally distributed, the Mann–Whitney U test was applied. For all tests, *p* < 0.05 was considered statistically significant.

## 3. Results and Discussion

The aim of presented study was to examine effects of substitution of raw, unprocessed CPS into the in the diet of broiler chickens. The diets used in our experiment were formulated to be isoprotein (Table 1). However, as CPS contains half as much protein as SBM (21% vs 46% CP in dry matter, Table 1) in order to obtain an equal content of crude protein in the diet, it was also necessary to change the share of cereals in prepared feed mixtures. Despite this, we prepared our mixtures to be also isonitrogenous and isoenergetic. However, as presented in previous works, despite the fact that experimental diets were not adjusted to other nutrients, the diet type did not have a significant effect on chicken weights (2320 ± 125 g and 2404 ± 170 g for the SBM and CPS group, respectively; *p* = 0.279) or feed conversion ratio (1.93 ± 0.34 kg/kg and 1.88 ± 0.44 kg/kg, for the SBM and the CPS groups, respectively; *p* = 0.863) [12,13]. A very recent experiment also showed that inclusion of raw CPS through the whole rearing period does not influence body weight gain and feed conversion ratio in turkeys [37]. Similarly to our experiment, the diets used in that study also differed in crude fat and crude fiber content.

**Figure 1:**
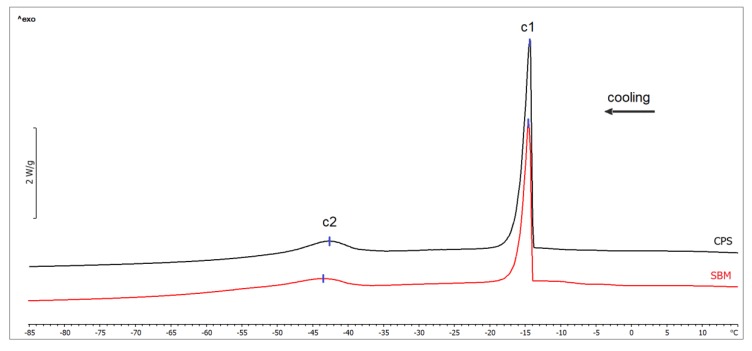
Differential scanning calorimetry (DSC) cooling thermograms of subcutaneous fat samples from broiler chickens fed soybean meal (SBM) and chickpea seeds (CPS). Exothermal processes are directed up.

In the examined adipose tissue samples, there were no statistically significant differences in the water content in the samples between examined groups (22.86 ± 3.76 g/100 g and 26.97 ± 4.22 g/100 g, for the SBM and CPS group, respectively; *p* = 0.059). Figure 1 shows the sample thermograms obtained during the crystallization of lipids in the examined subcutaneous fat samples. The results of determined thermal parameters are given in Table 2. The first exothermic peak in crystallization curve, marked as c1, corresponds to the freezing of the water present in subcutaneous fat tissue [5]. The freezing temperature did not differ between groups (*p* = 0.531). However, the net enthalpy was greater in the subcutaneous fat samples obtained from broilers in the CPS group (*p* = 0.029). The phase transition marked as c2 is associated with crystallization of lipids [29]. No statistically significant differences were found between the examined groups, either in the lipid crystallization temperature (*p* = 0.753) or in the net transition enthalpy (*p* = 0.434).

The available literature lacks data on the crystallization of subcutaneous fat of broilers, which does not allow any comparison of the results obtained in our study. There is one study in which broiler fat samples were analyzed using DSC, however there is no information on the broiler body part from which the analyzed samples were collected [34]. In this study, the fat crystallization temperature was found to be −41 °C, which is a result comparable to that obtained in our study, as the slight difference might be both due to the different content of unsaturated TAGs in the analyzed samples, or applied experimental protocol (sample preparation, heating rate).

**Figure 2:**
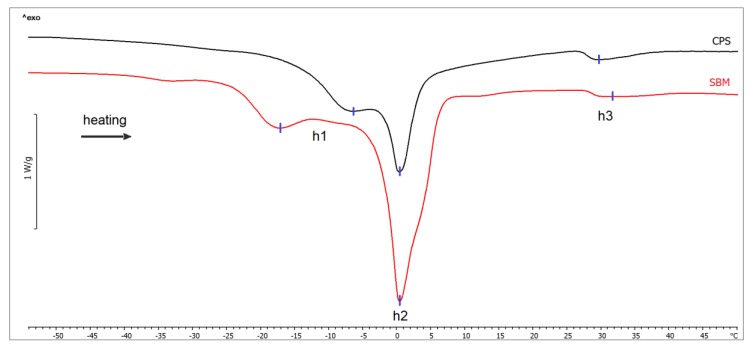
DSC heating thermograms of subcutaneous fat samples from broiler chickens fed soybean meal (SBM) and chickpea seeds (CPS). Exothermal processes are directed up.

Figure 2 shows the sample thermograms obtained during the melting of lipids in the examined subcutaneous fat samples. The results of determined thermal parameters are given in Table 3. During the heating of the frozen samples, three endothermic peaks were identified. The first, marked as h1, corresponds to the phase transition of the low-melting fraction of monounsaturated TAGs [30,35]. The peak temperature at maximum heat absorption was different for the SBM and CPS samples (*p* < 0.001). The position of the second peak, marked as h2, which corresponds to the phase transition of the high-melting fraction of the polyunsaturated TAGs [30,35], was the same for both groups (*p* = 0.458). However, the net transition enthalpy of all the unsaturated TAG fractions changed significantly, and was higher for the samples obtained from the chickens fed CPS (*p* = 0.007). The melting temperature of the saturated TAGs (peak h3) did not differ between groups (*p* = 0.064), however, the net transition enthalpy decreased in the CPS group (*p* < 0.001). Saturated TAGs make up only 15–18% of adipose tissue in broilers [36], so the observed peak associated with their melting is significantly lower than the peaks observed for all fractions of the unsaturated TAGs. The available literature only presents the results from DSC analyses of melting chickens’ abdominal fat [5]. The main melting temperature was determined at 0.68 °C [5], which corresponds to the location of the main peak of the fraction of low-melting monounsaturated TAGs observed in our study (0.34 ± 0.54 °C and 0.52 ± 0.35 °C, for SBM and CPS group, respectively).

Feeding has a significant impact on the fatty acid profile in adipose tissue in broilers, and it is known that the proportion of adipose tissue in the carcass is affected by the amount of protein in feed [2]. This study shows, for the first time, that also other feed ingredients affect the adipose tissue melting point, which is an important fat quality parameter. The process of melting of TAGs depends, among others, on the composition and the ratio of monounsaturated to saturated fatty acids [29,38]. SBM differs significantly from CPS in fatty acid profile [39,40]. Linoleic acid is the dominant fatty acid in SBM and CPS (about 50–55% for both). SBM has higher amounts of palmitic acid compared to CPS (13% and 9%, respectively), while higher amounts of palmitoleic acid can be found in CPS (0.30% and 0.13%, respectively). The biggest difference in fatty acid profile between SBM and CPS is observed in oleic acid content, which amount in CPS to twice as much as in SBM (32% and 12–16%, respectively) [39,40].

Table 4 shows fatty acid composition of the examined subcutaneous fat tissue samples, and some of the observed differences in the fatty acid content in tissue reflecting those found in CPS and SBM—while the content of linoleic acid did not differ between groups (*p* = 0.803), a higher content of palmitic acid was found in the adipose tissue of birds from the SBM group (decrease by 13%, from 22.97% to 20.37% in the SBM and CPS group, respectively; *p* < 0.001), and a higher content of oleic acid was found in the adipose tissue of birds from the CPS group (increase by 19%, from 35.99% to 42.81% in the SBM and CPS group, respectively; *p* < 0.001). Interestingly, a higher content of palmitoleic acid was found in the adipose tissue of birds from the SBM group (decrease by 13%, from by 1.99% to 1.72% in the SBM and CPS group, respectively; *p* < 0.001). In our study, the biggest difference was observed for linolenic acid, the proportion of which, although low in both groups, increased by 30% in the CPS group (from 1.06% to 1.38% in the SBM and CPS group, respectively; *p* < 0.001). While numerous mammalian-based experiments show that some fatty acids have specific effects on metabolic pathways, these aspects are still poorly known in poultry, however a higher content of palmitic acid suggests a higher lipogenic activity in birds from the SBM group [41].

When comparing the results of DSC analysis with FA composition of the samples, it can be noted that differences of net transition enthalpy of identified fractions of TAGs generally correspond to the differences in FA proportions in the subcutaneous fat tissue. The fat samples from the SBM group were characterized with a higher content of saturated FA (SFA, Table 4; *p* = 0.003) and these samples had higher net transition enthalpy of saturated TAGs (Table 3). Similarly, the higher content of monounsaturated FA (MUFA) was determined in the fat samples from the CPS group (Table 4; *p* < 0.001) and the higher net transition enthalpy of all unsaturated TAGs was observed (Table 3).

According to Bavelaar and Beynen [42], there is generally a weak relationship between oleic acid dietary content and level of oleic acid in broilers tissues and thermal properties of abdominal fat; on the contrary, Gallardo et al. [43] showed that the content of oleic acid in subcutaneous adipose tissue can change to the greatest extent, depending on its content in feed ingredients. While our results generally support the results by Gallardo et al., different fatty acid profiles of SBM and CPS may be not the only reason of observed differences in the FA profile and thermal properties of subcutaneous fat tissue, as dietary PUFA changes are generally more pronounced in meat fat that in subcutaneous fat [44]. Further, birds from the CPS group were also fed diets which were characterized with the lower content of methionine and lysine throughout the whole rearing period (Table 1), which were slightly below the recommended levels for meat-type chickens [45]. Both amino acids affect fat deposition in poultry. It has been shown that a deficiency of methionine leads to a significant increase in body fat in broiler chickens and turkeys [46,47]. It is assumed that methionine influences the processes of lipogenesis and lipolysis [4]. On the other hand, an increase in the lysine content in feed reduces fat accumulation by inhibiting lipogenesis [48]. However, when the lysine content in feed was above the recommended level, an increased accumulation of fat was observed. This might have been due to a lack of amino acid balance in the diet, which promotes the conversion of glucose into fatty acids [49].

Soy isoflavones have an effect on the physiological functions of various tissues and organs, including adipose tissue and the liver [50,51]. They increase the oxidation of fatty acids in the liver by increasing the expression of isotype γ of peroxisome proliferator-activated receptors (PPARγ), which regulate adipogenesis [52,53]. Although the effect of dietary supplements with soy isoflavones on fat gain in 52-day-old broiler chickens has not been demonstrated [54], there is no information available on the possible effect of isoflavonides on the TAG profile in adipose tissue.

The effect of protein source on adipose tissue in broiler chickens was also very recently investigated by Niu and coworkers [46]. They focused on the expression of lipid-related genes and serum metabolites in broiler chickens fed diets where SBM was partially replaced with fermented cottonseed meal (FCSM). They showed that even a partial replacement of protein source influences subcutaneous fat thickness and has an effect on lipid-related gene expression in abdominal and liver tissues, as well as lipid-related serum indices and metabolites. While the effects of FCMS on lipid metabolism might be associated with the probiotic action of FCSM, the mechanisms of the effect of other protein sources on lipid metabolism in chickens remain unclear. One of the proposed general mechanism assumes that regulation of fat deposition is achieved through interacting with hormone secretion, including insulin [55]. In birds, triglyceride synthesis takes place mainly in liver [1,6,56] and insulin can regulate liver lipid metabolism [57]. Some bioactive components derived from proteins, like β-casomorphin, a peptide derived from β-casein of milk, can modulate animals’ feed intake and nutrient utilization through changes in hormone secretion by controlling the gene expression of lipid metabolism [55].

Clarification of the physiological mechanism underlying chicken adipose tissue development can help also limit the accumulation of subcutaneous and abdominal fat deposition in the chicken, which is one of the main problems in the broiler industry [56,58]. Subcutaneous fat constitutes a major part of the fat in broiler carcass and would be most easily lost in processing if any change in thermal properties resulted in a melting behavior of the fat [59,60]. What is more, broiler chickens, due to their rapid growth, have become recently a unique nonmammalian animal model for metabolic disorders and obesity in humans [61,62,63], where subcutaneous fat tissue has been reported to act as a buffer which can limit the exposure of other tissues to excessive fatty acid flux that can lead to lipotoxicity [64]. For this reason, we believe that physical properties and fatty acid composition of chicken subcutaneous fat tissue should be extensively examined.

While our findings provide current evidence for how broiler chickens respond to a dietary inclusion of raw chickpea seeds, our work has some limitations that require comments. First, we did not perform an analysis of the composition of fatty acids in feeds. Second, the CPS diet did not match the SBM diet in terms of essential amino acids and dietary nutrients. Finally, the study should include analysis of the differences of the nutrient intake. In our opinion, all the above limitations do not diminish completely the novel aspect of our work and this trial design was necessary to establish whether or not subcutaneous fat tissue was directly affected by inclusion of raw, unprocessed CPS into broiler diets. Further, using a DSC technique, it was possible to relate each transition to the different TAG fractions forming adipose tissue, showing that the feed ingredients have an effect on structural properties of TAGs and indicating that adipose tissue differs in structure and composition, which was confirmed by chromatographic analysis. To the best of our knowledge, this is the first study of this type, making it a benchmark for reporting the effects of feed ingredients on thermal properties of subcutaneous fat tissue in broilers. However, further investigation is required to determine physiological implications of the observed changes and to look for any correlations between thermal properties and functional outcomes.

## 4. Conclusions

The results obtained in this study clearly indicate that differences in feed ingredients can affect both fatty acid composition and structural properties of subcutaneous adipose tissue in broiler chickens. Due to the lack of other studies in this area, the mechanisms responsible for these effects remain unknown, and require further research. Owing to numerous physiological functions of subcutaneous fat tissue, understanding these mechanisms can promote the use of alternative protein both in poultry and human nutrition.

## Figures and Tables

**Table 1:** Ingredients and nutrients content of control soybean meal (SBM) (as-fed basis) and experimental chickpea seed (CPS) diets.

	Starter (d. 1–21)		Grower (d. 22–35)		Finisher (d. 36–42)	
	SBM	CPS	SBM	CPS	SBM	CPS
**Ingredient (%)**						
Maize	10.00	10.00	10.00	10.00	15.00	10.00
Wheat	53.75	21.40	44.91	19.41	35.25	19.95
Soybean meal ^1^	28.65	-	21.50	-	19.40	-
Chickpea seeds ^2^	-	45.00	-	45.00	-	45.00
Triticale	-	10.00	10.00	10.00	15.00	10.00
Rapeseed meal	-	2.00	4.00	-	5.00	-
Soybean oil	2.40	2.40	4.40	4.40	5.20	5.20
Monocalcium phosphate	0.88	0.88	0.83	0.83	0.80	0.80
Limestone	1.35	1.35	1.31	1.31	1.30	1.30
Sodium bicarbonate	0.08	0.08	0.08	0.08	0.08	0.08
Sodium chloride	0.30	0.30	0.27	0.27	0.27	0.27
Fat-protein concentrate ^3^	1.00	1.00	1.00	1.00	1.00	1.00
Premix vita-min	0.50 ^I^	0.50 ^I^	0.50 ^II^	0.50 ^II^	0.50 ^III^	0.50 ^III^
Choline chloride	-	4.00	-	6.00	-	4.70
DL-methionine 99%	0.09	0.09	0.10	0.10	0.10	0.10
L-lysine HCl 78%	0.30	0.30	0.30	0.30	0.30	0.30
L-threonine 99%	0.50	0.50	0.50	0.50	0.50	0.50
Carbovet ^4^	0.20	0.20	0.30	0.30	0.30	0.30
**Analyzed composition ^5^**						
Crude protein, %	21.1	21.2	19.0	19.1	18.0	18.1
Crude fat, %	4.28	5.21	6.23	8.23	7.09	9.0
Crude fiber, %	3.12	1.32	3.34	1.23	3.37	1.24
Lysine, %	1.34	0.98	1.21	0.86	1.14	0.78
Methionine + Cysteine, %	0.97	0.82	0.88	0.65	0.90	0.61
Total Ca, %	0.93	0.83	0.91	0.82	0.82	0.81
Total P, %	0.69	0.51	0.69	0.45	0.68	0.44
**Calculated composition**						
ME, MJ/kg	12.4	12.5	12.9	13.0	13.1	13.1
Bioavailable P, %	0.44	0.35	0.42	0.34	0.41	0.33
Total Ca / bioavailable P	2.12	2.32	2.14	2.40	2.17	2.41

SBM, the group fed with soybean meal; CPS, the group fed with chickpea seeds. ^1^ 46% crude protein in dry matter. ^2^ 21% crude protein in dry matter. ^3^ 1 kg of fat-protein concentrate contains: 39% crude protein, 2% crude fat, 10.8 MJ metabolizable energy. ^4^ 90% airy charcoal in dry matter. ^5^ Detailed information regarding the chemical analyses of the diet are presented in [13]. ^I^ Vitamin–mineral premix per kilogram of starter: Mn 100 mg, Fe 40 mg, Cu 16 mg, I 1 mg, Se 0.15 mg, vit. A 15 000 IU, vit. B1 3 mg, vit. B2 8 mg, vit. B6 5 mg, vit. B12 0.016 mg, vit. D3 5 000 IU, vit. E 75 mg, vit. K3 4 mg, choline 1 800 mg, folic acid 2 mg, biotin 0.2 mg, nicotinic acid 60 mg, pantothenic acid 18 mg. ^II^ Vitamin–mineral premix per kilogram of grower: Mn 100 mg, Fe 40 mg, Cu 16 mg, I 1 mg, Se 0.15 mg, vit. A 12 000 IU, vit. B1 2 mg, vit. B2 6 mg, vit. B6 4 mg, vit. B12 0.016 μg, vit. D3 5 000 IU, vit. E 50 mg, vit. K3 3 mg choline 1 600 mg, folic acid 1.75 mg, biotin 0.2 mg, nicotinic acid 60 mg, pantothenic acid 18 mg. ^III^ Vitamin–mineral premix per kilogram of finisher: Mn 100 mg, Fe 40 mg, Cu 16 mg, I 1 mg, Se 0.15 mg, vit. A 12 000 IU, vit. B1 2 mg, vit. B2 5 mg, vit. B6 3 mg, vit. B12 0.011 μg, vit. D3 5 000 IU, vit. E 50 mg, vit. K3 2 mg, choline 1 600 mg, folic acid 1.5 mg, biotin 0.05 mg, nicotinic acid 35 mg, pantothenic acid 18 mg.

**Table 2:** DSC freezing parameters of subcutaneous fat of broiler chickens fed soybean meal and chickpea seeds.

Transition	Temperature T (°C)		*p*-value	Enthalpy ΔH (J/g)		*p*-value
	SBM	CPS		SBM	CPS	
Water freezing (c1)	−14.21 ± 1.36	−13.44 ± 1.86	0.361	22.91 ± 5.50	29.38 ± 5.10	0.029
Lipids crystallization (c2)	−43.25 ± 0.99	−42.98 ± 2.16	0.753	5.58 ± 0.95	6.15 ± 1.78	0.434

Data are mean values ± SD. SBM, the group fed with soybean meal (n = 8); CPS, the group fed with chickpea seeds (n = 8).

**Table 3:** DSC melting parameters of subcutaneous fat of broiler chickens fed soybean meal and chickpea seeds.

Transition	Temperature T (°C)		*p*-value	Enthalpy ΔH (J/g)		*p*-value
	SBM	CPS		SBM	CPS	
Low-melting fraction of monounsaturated TAGs (h1)	−6.76 ± 0.55	−15.81 ± 4.28	<0.001	84.64 ± 7.48	106.76 ± 18.42	0.007
High-melting fraction of polyunsaturated TAGs (h2)	0.34 ± 0.54	0.52 ± 0.35	0.458			
Melting of saturated TAGs (h3)	30.31 ± 1.03	29.43 ± 0.67	0.064	2.88 ± 0.34	1.80 ± 0.46	<0.001

Data are mean values ± SD. SBM, the group fed with soybean meal (n = 8); CPS, the group fed with chickpea seeds (n = 8). TAGs, triacylglycerols.

**Table 4:** Fatty acids proportions (%) in the subcutaneous fat tissue of broiler chickens fed soybean meal and chickpea seeds.

Fatty Acid	SBM	CPS	*p*-Value
Miristic (14:0)	0.33 ± 0.03	0.41 ± 0.16	0.175
Palmitic (16:0)	22.97 ± 0.25	20.37 ± 0.47	<0.001
Heptadecanoic (17:0)	0.11 ± 0.04	0.10 ± 0.03	0.611
Estearic (18:0)	4.86 ± 0.20	4.90 ± 0.50	0.835
Palmitoleic (16:1)	1.99 ± 0.17	1.72 ± 0.06	<0.001
Oleic (18:1)	35.99 ± 2.59	42.81 ± 1.35	<0.001
Linoleic (18:2)	20.82 ± 3.42	21.56 ± 4.01	0.803
Linolenic (18:3)	1.06 ± 0.04	1.38 ± 0.17	<0.001
Saturated (SFA)	28.27 ± 1.50	25.78 ± 1.21	0.003
Monounsaturated (MUFA)	37.98 ± 2.37	44.54 ± 1.72	<0.001
Polyunsaturated (PUFA)	21.88 ± 2.65	22.94 ± 3.11	0.475
SFA: MUFA:PUFA	1:1.3:0.7	1:1.7:0.9	

Data are mean values ± SD. SBM, the group fed with soybean meal (n = 8); CPS, the group fed with chickpea seeds (n = 8).

## References

[1] Fouad A.M., El-Senousey H.K. (2014). Nutritional factors affecting abdominal fat deposition in poultry: A review. Asian Australas. J. Anim. Sci..

[2] Alleman F., Michel J., Chagneau A.M., Leclercq B. (2000). The effects of dietary protein independent of essential amino acids on growth and body composition in genetically lean and fat chickens. Br. Poult. Sci..

[3] Adams K.A., Davis A.J. (2001). Dietary protein concentration regulates the mRNA expression of chicken hepatic malic enzyme. J. Nutr..

[4] Heydarpour F., Amini B., Kalantari S.S., Akbari A., Heydarpour P. (2007). Mean percentage of skin and visible fat in 10 chicken carcass weight. Int. J. Poult. Sci..

[5] Marx S.D., Siares J.M., Rrestes R.C., Schinitzler E., Oliviera C.S., Demiate I.M., Backes G.T., Steffens J. (2016). Influence of sex on the physical–chemical characteristics of abdominal chicken fat. Braz. J. Poult. Sci..

[6] Wang G., Kin W.K., Cline M.A., Gilbert E.R. (2017). Factors affecting adipose tissue development in chickens: A review. Poult. Sci..

[7] Tomaszewska E., Muszyński S., Dobrowolski P., Kwiecień M., Klebaniuk R., Szymańczyk S., Tomczyk A., Kowalik S., Milczarek A., Świetlicka I. (2018). The influence of dietary replacement of soybean meal with high–tannin faba beans on gut–bone axis and metabolic response in broiler chickens. Ann. Anim. Sci..

[8] Tomaszewska E., Dobrowolski P., Klebaniuk R., Kwiecień M., Tomczyk-Warunek A., Szymańczyk S., Kowalik S., Milczarek A., Blicharski T., Muszyński S. (2018). Gut–bone axis response to dietary replacement of soybean meal with raw low–tannin faba bean seeds in broiler chickens. PLoS ONE.

[9] Milczarek A., Osek M. (2019). Effectiveness evaluation of use of various protein feeds for broiler chicken feeding. Ann. Anim. Sci..

[10] Rutkowski A., Kaczmarek S.A., Hejdysz M., Nowaczewski S., Jamroz D. (2015). Concentrates made from legume seeds (*Lupinus Angustifolius*, *Lupinus Luteus* and *Pisum Sativum*) and rapeseed meal as protein sources in laying hen diets. Ann. Anim. Sci..

[11] Konieczka P., Czerwiński J., Jankowiak J., Ząbek K., Smulikowska S. (2019). Effects of partial replacement of soybean meal with rapeseed meal, narrow–leaved lupin, DDGS, and probiotic supplementation, on performance and gut microbiota activity and diversity in broilers. Ann. Anim. Sci..

[12] Muszyński S., Kwiecień M., Świetlicki M., Dobrowolski P., Tatarczak J., Gładyszewska B. (2018). Effects of replacing soybean meal with chickpea seeds in the diet on mechanical and thermal properties of tendon tissue in broiler chicken. Poult. Sci..

[13] Muszyński S., Tomaszewska E., Dobrowolski P., Kwiecień M., Wiącek D., Świetlicka I., Skibińska M., Szymańska-Chargot M., Orzeł J., Świetlicki M. (2018). Analysis of bone osteometry, mineralization, mechanical and histomorphometrical properties of tibiotarsus in broiler chickens demonstrates a influence of dietary chickpea seeds (Cicer arietinum L.) inclusion as a primary protein source. PLoS ONE.

[14] Józefiak D., Józefiak A., Kierończyk B., Rawski M., Świątkiewicz S., Długosz J., Engberg R.M. (2016). Insects–a natural nutrient source for poultry–a review. Ann. Anim. Sci..

[15] Józefiak A., Nogales-Mérida S., Mikołajczak Z., Rawski M., Kierończyk B., Mazurkiewicz J. (2019). The utilization of full-fat insect meal in rainbow trout (*Oncorhynchus mykiss*) nutrition: The effects on growth performance, intestinal microbiota and gastro-intestinal tract histomorphology. Ann. Anim. Sci..

[16] Lei X.J., Kim T.H., Park J.H., Kim I.H. (2019). Evaluation of supplementation of defatted black soldier fly (*Hermetia illucens*) larvae meal in beagle dogs. Ann. Anim. Sci..

[17] Bampidis V., Christodoulou V. (2011). Chickpeas (*Cicer arietinum* L.) in animal nutrition: A review. Anim. Feed Sci. Technol..

[18] Trębacz H., Szczęsna A., Arczewska M. (2018). Thermal stability of collagen in naturally ageing and in vitro glycated rabbit tissues. J. Therm. Anal. Calorim..

[19] Zhang W., Liu L., Xiong Y., Liu Y., Yu S., Wu C., Guo W. (2018). Effect of in vitro storage duration on measured mechanical properties of brain tissue. Sci. Rep..

[20] Wiegand N., Nőt L.G., Patczai B., Lőrinczy D. (2017). The role of differential scanning calorimetry in the diagnostics of musculoskeletal disease. EC Orthop..

[21] Vega S., Garcia-Gonzalez M., Lanas A., Velazquez-Campoy A., Abian O. (2015). Deconvolution analysis for classifying gastric adenocarcinoma patients based on differential scanning calorimetry serum thermograms. Sci. Rep..

[22] Ferencz A., Nedvig K., László E., Magyarlaki T., Lőrinczy D. (2011). DSC examination of kidney tissue following warm ischemia and reperfusion injury. Thermochim. Acta.

[23] Szabó I., Bognár G., Kereskai L., Szász K., Lőrinczy D. (2007). Differential scanning calorimetric and histological examinations of the long head of the biceps in cadavers. J. Therm. Anal. Calorim..

[24] Chaudhury S., Holland C., Porter D., Tirlapur U.K., Vollrath F., Carr A.J. (2011). Torn human rotator cuff tendons have reduced collagen thermal properties on differential scanning calorimetry. J. Orthop. Res..

[25] Stangierski J., Tomaszewska-Gras J., Baranowska H.M., Krzywdzińska-Bartjowiak M., Konieczny P. (2019). The effect of deep pectoral myopathy on the properties of broiler chicken muscles characterised by selected instrumental techniques. Eur. Food Res. Technol..

[26] Wattanachant S., Benjakul S., Ledward D.A. (2005). Microstructure and thermal characteristics of Thai indigenous and broiler chicken muscles. Poult. Sci..

[27] Voutila L., Ruusunen M., Jouppila K., Puolanne E. (2009). Thermal properties of connective tissue in breast and leg muscles of chickens and turkeys. J. Sci. Food Agric..

[28] Saadi S., Ariffin A.A., Ghazali H.M., Miskandar M.S., Boo H.C., Abdulkarim S.M. (2012). Application of differential scanning calorimetry (DSC), HPLC and pNMR for interpretation primary crystallization caused by combined low and high melting TAGs. Food Chem..

[29] Yılmaz M.T., Karakaya M., Aktas N. (2010). Composition and thermal properties of cattle fats. Eur. J. Lipid Sci. Technol..

[30] Traffano-Schiffo M.V., Castro-Giraldez M., Colom R.J., Fito P.J. (2017). Development of a spectrophotometric system to detect white striping physiopathy in whole chicken carcasses. Sensors.

[31] Muszyński S., Tomaszewska E., Kwiecień M., Dobrowolski P., Świetlicka I., Tanaś W., Sołowiej B., Ejtel M., Szcześniak E., Tomczyk-Warunek A. (2019). The dietary inclusion of chickpea seeds (*Cicer arietinum* L.) influences thermal properties of muscle proteins but not the texture of drumstick muscle in broiler chickens. Braz. J. Poult. Sci..

[32] Muszyński S., Tomaszewska E., Kwiecień M., Dobrowolski P. (2019). The effect of raw chickpea seeds in broiler chickens feed on the thermal properties of collagen in the skin tissue. Pasze Przem..

[33] Blicharski T., Tomaszewska E., Dobrowolski P., Hułas-Stasiak M., Muszyński S. (2017). A metabolite of leucine (β–hydroxy–β–methylbutyrate) given to sows during pregnancy alters bone development of their newborn offspring by hormonal modulation. PLoS ONE.

[34] Dahimi O., Rahim A.A., Abdulkarim S.M., Hassan M.S., Hashari S.B.T., Mashitoh A.S., Saadi S. (2014). Multivariate statistical analysis treatment of DSC thermal properties for animal fat adulteration. Food Chem..

[35] Marikkar J.M.N., Ghazali H.M., Che Man Y.B., Lai O.M. (2002). The use of cooling and heating thermograms to monitoring of tallow, lard and chicken fat adulterations in canola oil. Food Res. Int..

[36] Folch J.M., Lees M., Stanley G.H.S. (1957). A simple method for the isolation and purification of total lipids from animal tissues. J. Biol. Chem..

[37] Ciurescu G., Vasilachi A., Grosu H. (2020). Efficacy of microbial phytase on growth performance, carcass traits, bone mineralization, and blood biochemistry parameters in broiler turkeys fed raw chickpea (*Cicer arietinum* L., cv. *Burnas*) *diets*. J. App. Poult. Res..

[38] Knothe G., Dunn R.O. (2009). A comprehensive evaluation of the melting points of fatty acids and esters determined by differential scanning calorimetry. J. Am. Oil Chem. Soc..

[39] Lee J.W., Kil D.Y., Keever B.D., Killefer J., McKeith F.K., Sulabo R.C., Stein H.H. (2013). Carcass fat quality of pigs is not improved by adding corn germ, beef tallow, palm kernel oil, or glycerol to finishing diets containing distillers dried grains with solubles. J. Anim. Sci..

[40] Jukanti A.K., Gaur P.M., Gowda C.L., Chibbar R.N. (2012). Nutritional quality and health benefits of chickpea (*Cicer arietinum* L.): A review. Br. J. Nutr..

[41] Watkins B.A. (1991). Importance of essential fatty acids and their derivatives in poultry. J. Nutr..

[42] Bavelaar F.J., Beynen A.C. (2003). Relationships between dietary fatty acid composition and either melting point or fatty acid profile of adipose tissue in broilers. Meat Sci..

[43] Gallardo M.A., Perez D.D., Leighton F.M. (2012). Modification of fatty acid composition in broiler chickens fed canola oil. Biol. Res..

[44] Diaw M.T., Dieng A., Mergeai G., Dotreppe O., Youssouf I., Hornick J.L. (2010). Effect of groundnut cake substitution by glandless cottonseed kernels on broilers production: Animal performance, nutrient digestibility, carcass characteristics and fatty acid composition of muscle and fat. Int. J. Poult. Sci..

[45] Smulikowska S., Rutkowski A. (2018). Recommended Allowances and Nutritive Value of Feedstuffs. Poultry Feeding Standards.

[46] Niu J.L., Zhang J., Wei L.Q., Zhang W.J., Nie C.X. (2019). Effect of fermented cottonseed meal on the lipid-related indices and serum metabolic profiles in broiler chickens. Animas.

[47] Moran E.T. (1994). Response of broiler strains differing in body fat to inadequate methionine: Live performance and processing yields. Poult. Sci..

[48] Murawska D., Kubińska M., Gesek M., Zduńczyk Z., Brzostowska U., Jankowski J. (2018). The effect of different dietary levels and sources of methionine on the growth performance of turkeys, carcass and meat quality. Ann. Anim. Sci..

[49] Grisoni M.L., Uzu G., Larbier M., Geraert P.A. (1991). Effect of dietary lysine on lipogenesis in broilers. Reprod. Nutr. Dev..

[50] Nasr J., Kheiri F. (2012). Effects of lysine levels of diets formulated based on total or digestible amino acids on broiler carcass composition. Rev. Bras. Cienc. Avıcola.

[51] Orgaard A., Jensen L. (2008). The effects of soy isoflavones on obesity. Exp. Biol. Med..

[52] Takahashi Y., Ide T. (2008). Effects of soy protein and isoflavone on hepatic fatty acid synthesis and oxidation and mRNA expression of uncoupling proteins and peroxisome proliferator–activated receptor γ in adipose tissues of rats. J. Nutr. Biochem..

[53] Wang Y., Mu Y., Li H., Ding N., Wang Q., Wang Y., Wang S., Wang N. (2008). Peroxisome proliferator–activated receptor gamma gene: A key regulator of adipocyte differentiation in chickens. Poult. Sci..

[54] Payne R., Bidner T., Southern L., McMillin K. (2001). Dietary effects of soy isoflavones on growth and carcass traits of commercial broilers. Poult. Sci..

[55] Chang W.H., Zheng A.J., Chen Z.M., Zhang S., Cai H.Y., Liu G.H. (2019). β-Casomorphin increases fat deposition in broiler chickens by modulating expression of lipid metabolism genes. Animal.

[56] Bai S., Wang G., Zhang W., Zhang S., Rice B.B., Cline M.A., Gilbert E.R. (2015). Broiler chicken adipose tissue dynamics during the first two weeks post-hatch. Comp. Biochem. Physiol. A.

[57] Dong X.Y., Tang S.Q. (2010). Insulin-induced gene: A new regulator in lipid metabolism. Peptides.

[58] Guo L., Sun B., Shang Z., Leng L., Wang Y., Wang N., Li H. (2011). Comparison of adipose tissue cellularity in chicken lines divergently selected for fatness. Poult. Sci..

[59] Husbands D.R. (1972). The effect of dietary copper on the composition of adipose tissue triglycerides in the broiler chicken. Br. Poult. Sci..

[60] Hrdinka C., Zollitsch W., Knaus W., Lettner F. (1996). Effects of dietary fatty acid pattern on melting point and composition of adipose tissues and intramuscular fat of broiler carcasses. Poult. Sci..

[61] Wang G., McConn B.R., Liu D., Cline M.A., Gilbert E.R. (2017). The effects of dietary macronutrient composition on lipid metabolism associated factor gene expression in the adipose tissue of chickens are influenced by fasting and refeeding. BMC Obes..

[62] Resnyk C.W., Carré W., Wang X., Porter T.E., Simon J., Le Bihan-Duval E., Duclos M.J., Aggrey S.E., Cogburn L.A. (2017). Transcriptional analysis of abdominal fat in chickens divergently selected on bodyweight at two ages reveals novel mechanisms controlling adiposity: Validating visceral adipose tissue as a dynamic endocrine and metabolic organ. BMC Genom..

[63] De Almeida Mallmann B., Martin E.M., Soo Kim K., Calderon-Apodaca N.L., Baxter M.F.A., Latorre J.D., Hernandez-Velasco X., Paasch-Martinez L., Owens C.M., Tellez-Isaias G. (2019). Evaluation of bone marrow adipose tissue and bone mineralization on broiler chickens affected by wooden breast myopathy. Front. Physiol..

[64] Frayn K. (2002). Adipose tissue as a buffer for daily lipid flux. Diabetologia.

